# Investigation of cell line specific responses to pH inhomogeneity and consequences for process design

**DOI:** 10.1002/elsc.202000034

**Published:** 2020-07-21

**Authors:** Katrin Paul, Thomas Hartmann, Christoph Posch, Dirk Behrens, Christoph Herwig

**Affiliations:** ^1^ Institute of Chemical Environmental and Bioscience Engineering TU Wien Vienna Austria; ^2^ Christian Doppler Laboratory for Mechanistic and Physiological Methods for Improved Bioprocesses TU Wien Vienna Austria; ^3^ Sandoz GmbH Schaftenau Austria

**Keywords:** 2‐compartment system, CHO, inhomogeneities, large‐scale, pH excursions, scale‐down

## Abstract

With increasing bioreactor volumes, the mixing time of the reactor increases as well, which creates an inhomogeneous environment for the cells. This can result in impaired process performance in large‐scale production reactors. Particularly the addition of base through the reactor headspace can be problematic, since it creates an area, where cells are repeatedly exposed to an increased pH. The aim of this study is to simulate this large‐scale phenomenon at lab‐scale and investigate its impact. Two different cell lines were exposed to pH amplitudes of a maximal magnitude of 0.05 units (pH of 6.95). Both cell lines showed similar responses, like decreased viable cell counts, but unaffected lactate levels. However, cell line B showed an initially increased specific productivity in response to the introduced amplitudes, whereas cell line A showed a consistently lower specific productivity. Furthermore, the time point at which base addition is started influences the impact, which pH amplitudes have on process performance. When pH control was started earlier in the process, maximal viable cell counts decreased and the lactate metabolic shift was less pronounced. These results show that the potential negative impact of pH amplitudes can be minimized by strategic process design.

**Abbreviations**
1/2‐CS1/2: Compartment system
CHO: Chinese Hamster Ovary
qmAb: Specific mAb production rate
VCC: Viable cell concentration

## 1 INTRODUCTION

The demand for biopharmaceuticals is steadily increasing and it has been predicted that approximately 50% of the biologics will continue to be produced in large‐scale bioreactors with volumes of at least 5000 L [1]. Bioreactor volumes in cell culture currently reach up to 25000 L and although production at large scale is more economic, there can be challenges associated with the scale‐up of a process. An increase in mixing time is always associated with larger reactor volumes and it can result, for example, in pH gradients [2, 3, 4, 5]. This difference in scales can have no impact on a process [6, 7], but it can also negatively affect process performance [8, 9, 10]. It has been shown in a case study, that shake flasks can demonstrate a better consistency in process performance with a large‐scale reactor (15,000 L) than a 3 L lab‐scale bioreactor, highlighting the impact different reactor scales can have on process performance. The main factors for the discrepancy between the scales were identified as differences in pH and CO_2_ [11]. While increased CO_2_ levels have been consistently shown to negatively impact process performance [12, 13, 14, 15], less is known about the impact of pH inhomogeneity on process performance. Due to the addition of base through the headspace of the large‐scale reactor, an area with an increased pH can form [4, 16]. A challenge for pH inhomogeneity studies in unmodified bioreactors is an increase in osmolality, which is a result of the addition of large amounts of base to introduce pH excursions [17]. 2‐Compartment Systems (2‐CS), which require a recirculation of the cells between multiple bioreactors, can on the other hand struggle with effects introduced by the pump [18]. The response to a single shift, as well as a single perturbation in pH has however been studied. It has been shown that a change in pH from 7.0 to 7.8 or 9.0 rapidly increases the intracellular pH of Chinese Hamster Ovary (CHO) cells [19]. Hybridoma cells have been shown robust to single pH excursion and pH shifts [20]. However, base addition at large‐scale would cause frequent pH excursions for only short periods of time [4, 21, 22]. Recent work has shown that the exposure of CHO cells to reoccurring pH amplitudes of an average magnitude of 7.3 already impairs process performance and inhibits the lactate metabolic shift [23]. Due to inherent differences of the various CHO cell lineages, the response to pH inhomogeneity may however vary from cell line to cell line [24].

Therefore, this study focuses on the investigation of differences between the cell line lineage specific response to pH inhomogeneity. Since previous work has shown a negative impact on process performance, when cells were introduced to pH amplitudes of 0.4 pH units [23], this study investigates amplitudes of a maximal magnitude of 0.1 pH units. Both cell lines were exposed to similar pH excursions in 2‐CSs, where base addition started at the same time point for all experiments. However, base addition started later in the previous study [23], therefore the impact of the starting time point of pH correction on process performance was also investigated. Furthermore, submerse base addition has been suggested as a solution to gradients occurring at large‐scale by improving mixing [4, 25]. The impact on cells has however not been evaluated and is investigated here as well.

## 2 MATERIALS AND METHODS

### 2.1 Cell line, preculture, and cultivation conditions

CHO suspension cell line A, producing mAb A and cell line B, producing mAb B, were cultivated in chemically defined medium. Both cell lines were derived from different CHO lineages. Preculture of the cells was performed in shake flasks at 10% pCO_2_, 36.5°C and 143 rpm to expand the cells before inoculation. Bioreactors were inoculated to achieve an initial cell density of 5 × 10^5^ cells mL^−1^.

**PRACTICAL APPLICATION**

Increasing mixing times in large‐scale bioreactors can result in the formation of gradients. Exposure of different cell lines to even minor pH amplitudes resulted in a negatively affected process performance. The overall response of the cells to the repeated exposure to pH excursions appears to be cell lysis, revealing an only limited adaptation ability of the cells. This was reflected in higher cell densities, as well as improved viability of the cells, when base addition was postponed. These results show, that even in a well‐mixed lab‐scale bioreactor, base addition presents a stress factor for the cells. The sensitivity of the cells not only to pH excursions, but to base addition in itself shows the importance of process design as a tool to minimize negative effects on process performance.

1‐compartment system (1‐CS) experiments were conducted in unmodified 3 L bioreactors (Labfors, Infors, Bottmingen, Switzerland) in which dissolved oxygen (dO_2_) (VisiFerm, Hamilton, Franklin, MA, USA) and pH (EasyFerm, Hamilton, Franklin, MA, USA) were monitored with inline probes. pCO_2_ was monitored with an offgas sensor (BlueInOne, Bluesens, Herten, Germany). dO_2_, pCO_2_ and pH were independently controlled. pH control was performed with either 0.5 M NaOH or 3% H_3_PO_4_, once the pH reached a value of 6.90. The dO_2_ (40%) and pCO_2_ (10%) were kept constant by the addition of O_2_ and CO_2_ respectively. Temperature was initially set to 36.50°C and shifted to 33°C, once a cell density of 25 × 10^5^ cells mL^−1^ (cell line A) or 35 × 10^5^ cells mL^−1^ was reached. Feed A was continually fed from day 4 (cell line A) or day 5 (cell line B) and Feed B started at day 6 (cell line A) or day 7 (cell line B) of the cultivation. Both feeds contain mixtures of different amino acids, which were individually optimized for both cell lines. Glucose was fed continually after its concentration dropped below 2 g L^−1^ to reestablish glucose levels of 2 g L^−1^. The 2‐CSs were setup according to previously published work [23]. They consist of a bioreactor vessel with added outlet at the bottom and a bypass for the recirculation of the cells. When the pH in the bioreactor drops beneath the dead band (0.02 pH units), base is added directly in the bypass. This creates a zone with increased pH throughout the bypass. Only 7.5% of the total cell population is exposed to the pH amplitudes, therefore mimicking the base addition zone of a large‐scale reactor. All experiments were performed once.

### 2.2 Analytical methods

Viable cell counts (VCC), dead cell counts, and cell size were determined with the Cedex HiRes automatic picture Analyzer (Roche, Mannheim, Germany). Lactate was measured with the Cedex Bio HT Analyzer (Roche, Mannheim, Germany). Concentration of the mAbs was measured by HPLC (Ultimate 3000, Dionex, Sunnyvale, CA, USA) with a protein A sensor cartridge (Applied Biosystems, Bleiswijk, Netherlands). Apoptotic cells and PI positivte/Annexin negative particles were determined with Annexin V Alexa Fluor™ 488 and Propidium Iodide (Thermo Fisher Scientific, Waltham, MA, USA). Cells were treated according to Kumar et al. except for the direct incubation of the cells in the staining solution, without prior washing of the cells [26].

### 2.3 Estimation of the product formation rate

The specific mAb production rate (q_mAb_) was approximated by

(1) $$\;q_{mAb}=\frac{{\left[mAb\right]}_{2}-\;{\left[mAb\right]}_{1}}{\left({\left[IVCD\right]}_{2}-{\left[IVCD\right]}_{1}\right)}$$

(2) $$\begin{array}{ccc} {\left[IVCD\right]}_{2} & = & \frac{\left({\left[VCC\right]}_{2}\cdot \;V_{2}+{\left[VCC\right]}_{1}\cdot \;V_{1}\right)}{2}\\ & & \cdot \;\left(t_{2}-t_{1}\right)+{\left[IVCD\right]}_{1} \end{array}$$

## 3 RESULTS AND DISCUSSION

### 3.1 Cell line specific impact of pH inhomogeneity on process performance

It has previously been shown that the exposure of cells to amplitudes of only 0.4 pH units already negatively impacts process performance. Furthermore, the number of exposed cells or the amount of added base was identified as the main culprits for impaired process performance [23]. Here the impact of smaller pH amplitudes (magnitude of 0.05 units) and of a narrow pH control without the introduction of amplitudes are investigated for cell lines A and B. The negative control is a 1‐CS, since comparability between the 1‐CS and 2‐CS has been previously established [23]. Additionally, cell line B was exposed to medium amplitudes (magnitude of 0.1 units).

**FIGURE 1:**
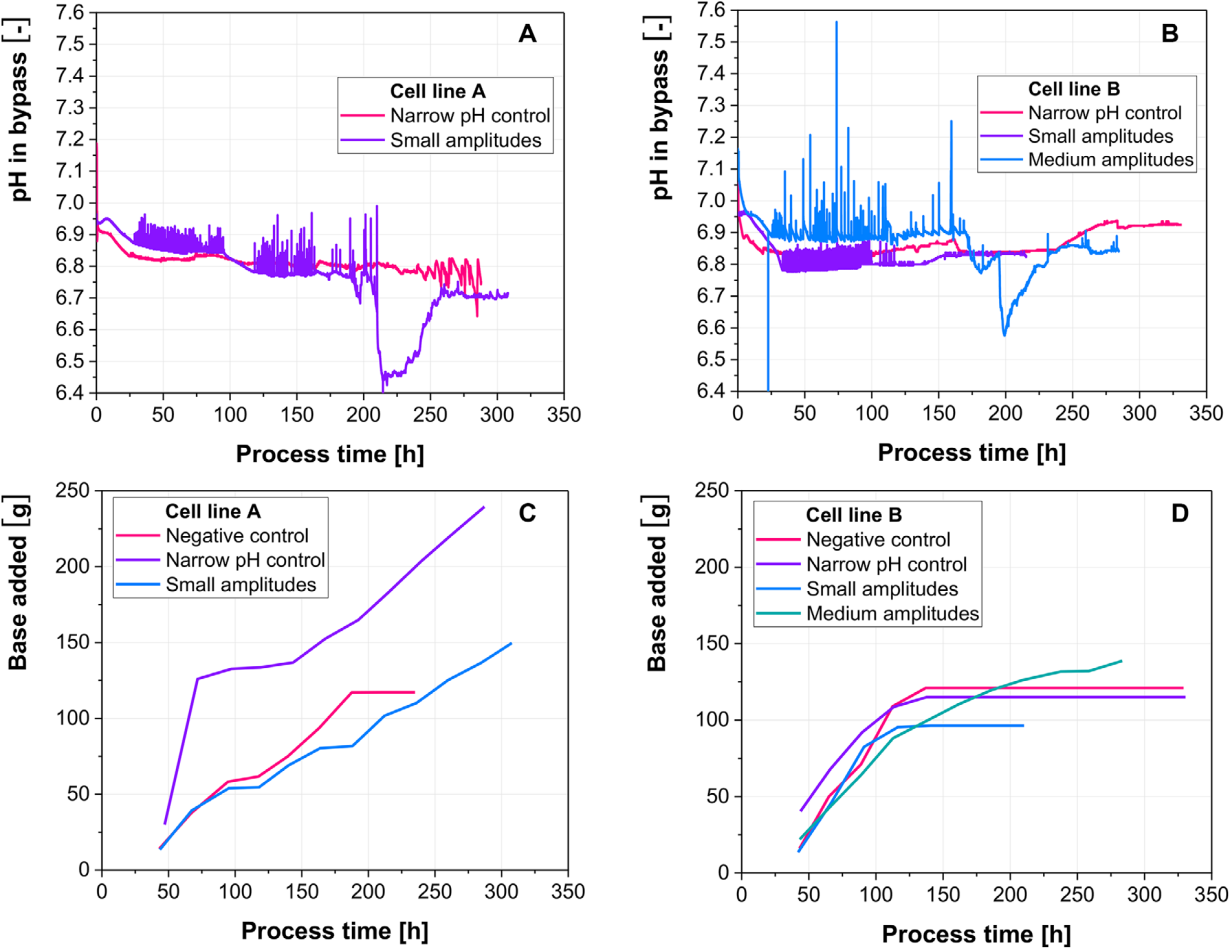
(A‐B) pH trajectories in the bypass of the 2‐CS for narrow pH control (no amplitudes are introduced), small amplitudes (0.05 pH units) and medium amplitudes (0.1 pH units). (C‐D) Amounts of base which were added throughout the cultivations

**FIGURE 2:**
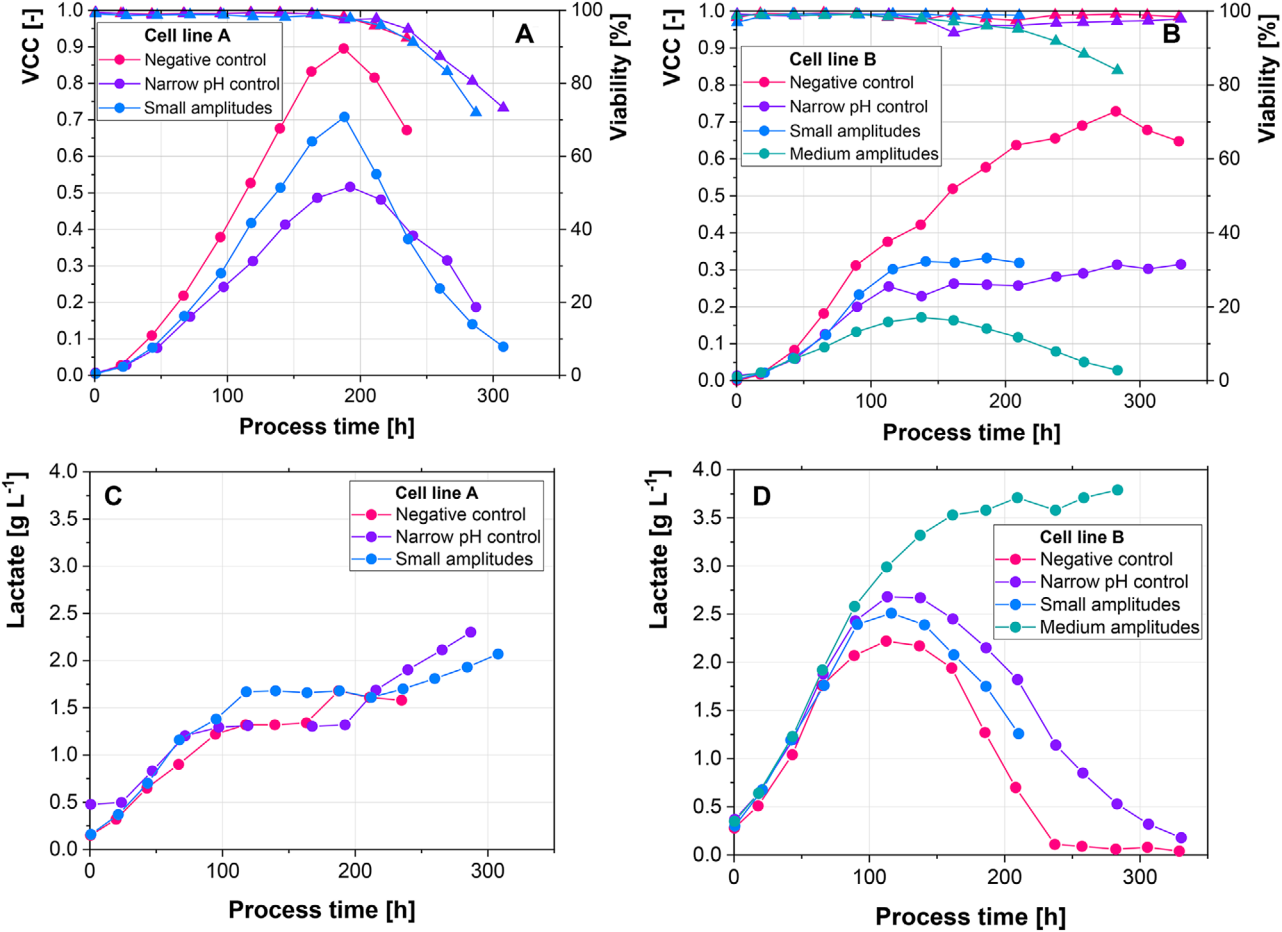
(A‐B) Viable cell concentration (dots) and viability (triangles) trajectories. (C‐D) Absolute lactate levels for the different introduced amplitudes

Figure 1A,B show the pH profiles in the bypass of the 2‐CS. Base was added to cultivations of cell line A continuously, except for the negative control, where base addition ceased around 180 h. Cell line B required acid addition after approximately 140 h, except for the experiment, where medium amplitudes (0.1 pH units) were introduced (Figure 1C,D). The sharp drops in pH for the small amplitudes (0.05 pH units) of cell line A and the medium amplitudes (0.1 pH units) of cell line B are likely related to cell lysis. Since base is added into the bypass right before the pH probe, the remains of lysed cells, particularly DNA, could be sticking to the probe and cause the drop in pH. This is in good agreement with Figure 2A,B, since the drops are correlated with the steepest decreases in VCC for both cell lines.

**FIGURE 3:**
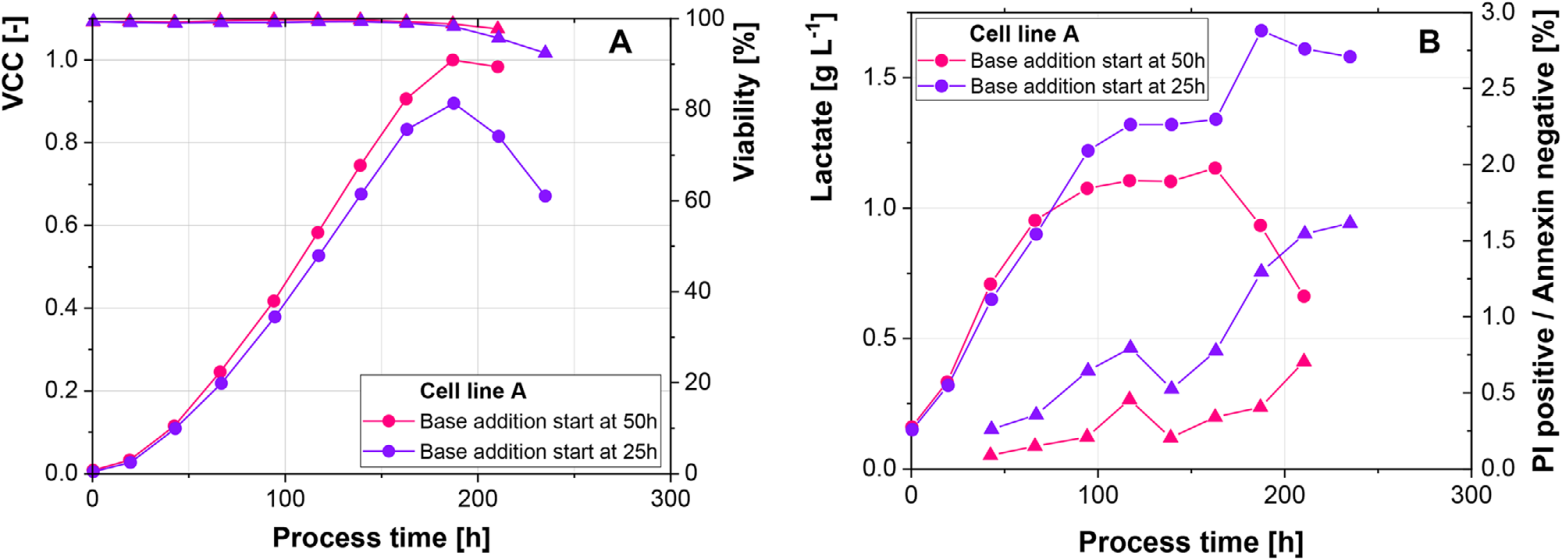
(A) Viable cell concentration (dots) and viability (triangles) trajectories. (B) Absolute lactate levels (dots) and percentage of PI positive/Annexin negative cells (triangles) for different time points of the start of pH control

Both cell lines show a decreased maximal VCC, when exposed to small amplitudes (0.05 pH units) and narrow pH control (no pH amplitudes) (Figure 2A,B). The decrease is more pronounced for cell line B, where only less than 50% of the maximal VCC were reached. Cell line A, which has shown only an 18% decrease in maximal VCC in a previous report [23], shows a decrease in maximal VCC of 22% when small amplitudes (0.05 pH units) are introduced and of 44% for the narrow pH control (no pH amplitudes). However, base addition was started earlier in this study, to achieve similar starting points for both cell lines. This indicates that the time point, when pH control is started is relevant to the impact of the pH amplitudes on process performance. The difference between both starting points for pH correction was assessed in 1‐CS experiments and is discussed later (Figure 3).

**FIGURE 4:**
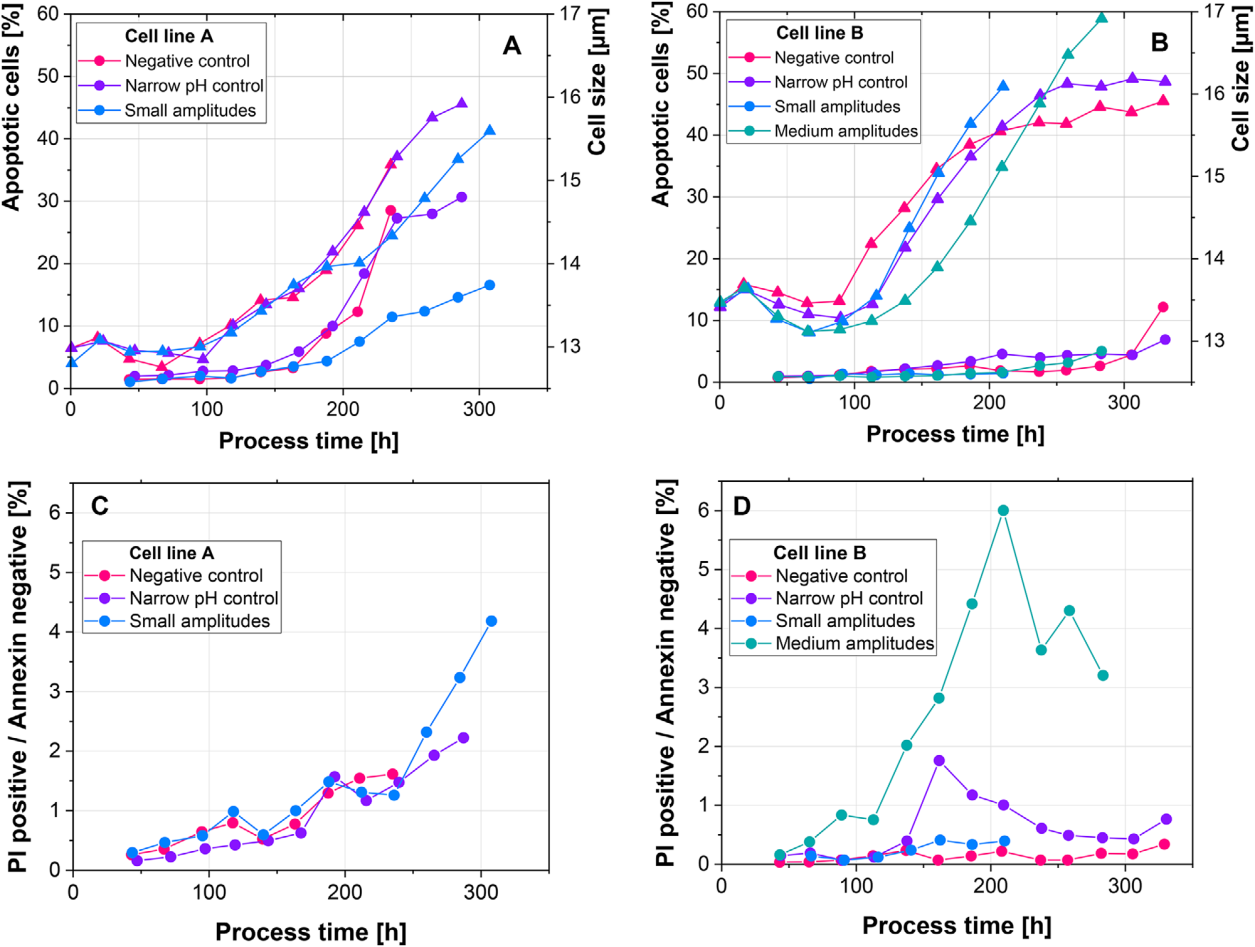
(A‐B) Percentage of apoptotic cells (dots) and cell size (triangles) over the process time. (C‐D) Percentage of PI positive/Annexin negative particles

Cell viabilities are similar for cell line A, while the medium amplitudes (0.1 pH units) of cell line B caused a premature decline in viability. Furthermore, when pH control was narrow (no pH amplitudes) for cell line B, viability dropped after 160 h and started to slowly increase again, eventually reaching similar values as the control cultivation. Since a spike in the amount of PI positive and Annexin negative cells (Figure 4D) correlates with the drop in viability, a mere measurement error is unlikely. This suggests that upon the cessation of base addition, the cells are able to recover from the base addition. Narrow pH control (no pH amplitudes) resulted in worse process performance than the small amplitudes (0.05 pH units), although less base was added with each pH correction. This suggests that by increasing the frequency of base addition, although less base is added at each point, more cells are affected by the base. The amounts of base which were added in total confirm this hypothesis. For cell line A, a total of 240 g of base was added, when the pH was controlled narrowly (no pH amplitudes), while only 150 g were added, when small amplitudes (0.05 pH units) were introduced. Similarly, 120 g were added for cell line B during narrow pH control in comparison to 100 g for small amplitudes. However, when medium amplitudes were introduced, less base was initially added (Figure 1D). This suggests that a more severe insult to less cells is more detrimental than a lesser insult to more cells. Overall the combination of the number of exposed cells with the strength of the insult appears to determine the overall impact on process performance. Absolute lactate levels, as well as specific lactate production/consumption rates, are similar between the different experiments (Figure 2C,D). Only the introduction of medium amplitudes (0.1 pH units) to cell line B showed increased lactate levels, as well as the absence of the metabolic shift from lactate production to lactate consumption. Since lactate metabolism has been shown to be a process indicator, as well as a predictor of final process outcomes, the absence of the lactate metabolic shift indicates that the exposure of cells to amplitudes of only 0.1 pH units has already the potential to negatively impact process performance [27, 28].

**FIGURE 5:**
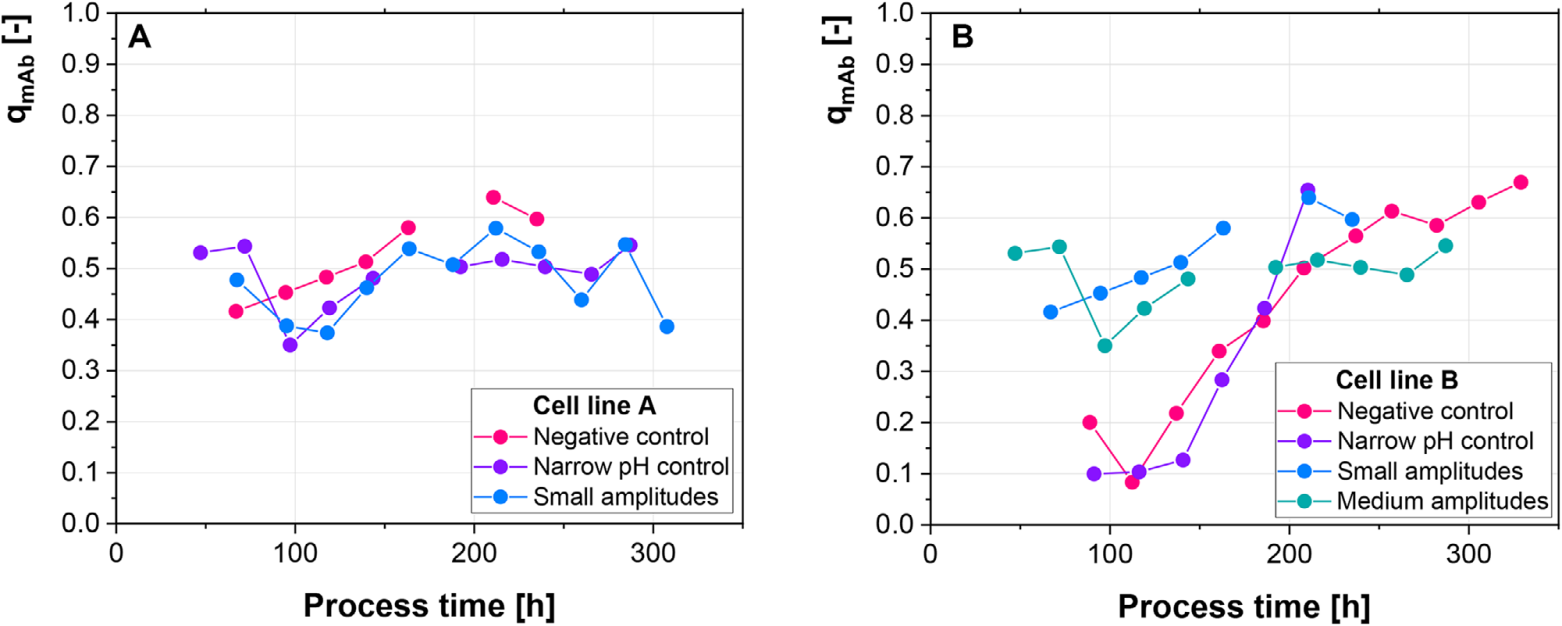
(A‐B) Specific mAb production rate of both cell lines

The qmAb is differently affected in both cell lines (Figure 5). While cell line A shows an almost consistently higher productivity of the negative control, cell line B shows a higher productivity for the medium (0.1 pH units) and small amplitudes (0.05 pH units) up until 200 h of the process. Similar to the increase in productivity in response to high osmolality [29, 30, 31], the introduced pH amplitudes could present non‐ideal growth conditions, which increase the specific productivity of the cells. Furthermore, a recent study showed that different CHO cell line lineages either have a preference for biomass synthesis or mAb productivity [24]. These inherent differences between the cell lineages could potentially be affecting their adaptation capabilities.

In terms of cell size, cell line A shows similar trajectories for the negative control and narrow pH control, while the small amplitudes resulted in smaller cells after 200 h (Figure 4A). Cell line B shows consistently smaller cells for all scenarios in comparison to the negative control until 150 h (Figure 4B). After this point cell size increases more rapidly in the 2‐CS and at the end of the process the negative control shows the smallest cells. Increased cell specific productivity has been correlated to increased cell size and therefore volume of the cells [32, 33, 34]. However, cell line B shows a lower cell size for the cells with higher productivity, indicating a different mechanism behind the increased initial productivity.

Stressful conditions like hyperosmotic stress, nutrient limitation and ammonia concentration have been correlated to increased levels of apoptotic cells [35, 36, 37]. However, neither cell line shows increased levels of apoptosis (Figure 4A,B). Cell line A even shows approximately 15% less apoptotic cells, when small amplitudes (0.05 pH units) are introduced, in comparison to the negative control. Furthermore, the lower percentage of apoptotic cells correlates with a decreased cell size. These results further point towards the limited capacity of the cells to adapt to the frequent, but short insults caused by base addition. This is further highlighted by the number of PI positive/Annexin negative cells (Figure 4C,D). Dead cells are positive for PI, due to a leaky cell membrane, and positive for Annexin, due to the displacement of phosphatidyl serine (PS) to the outer membrane [38]. Particles which are positive for PI and negative for Annexin are stained solely for their DNA content. Cell line B shows particularly high levels of this subpopulation, when medium amplitudes are introduced (Figure 4D). Furthermore, levels increase, when pH is controlled narrowly (no pH amplitudes), but decline, when base addition ceases at 140 h. Therefore, it appears that the cells disintegrate, rather than adapt, when they are exposed to pH inhomogeneity. This further shows the importance to avoid even rather small pH excursions at large‐scale.

### 3.2 Effect of starting point for pH control on process performance

Since higher pH amplitudes from a previous study [23] resulted in a lesser decrease of the maximal VCC of cell line A, different starting points for pH correction were investigated in a 1‐CS experiment. Base addition was started at 25 h in this study and at 100 h in the previous study. To correct for the lower inoculation cell densities in the previous study, pH control was started at a similar VCC, which was reached around 50 h in this study.

An earlier start of pH control resulted in a slightly decreased VCC, as well as a steep VCC decrease, after the maximal VCC was reached (Figure 3A). This is in alignment with the higher percentage of PI positive/Annexin negative particles (Figure 3B). Furthermore, a pronounced lactate metabolic shift is only occurring, when base addition is postponed. These results indicate that even in a well‐mixed lab‐scale bioreactor, the addition of base presents a potential stress for the cells. Since the temperature shift was performed at 50 h, delayed base addition affected the cells only after the exponential growth phase. It is well established for microbial cells, that stress resistance and growth are inversely correlated [39, 40, 41]. Since the decrease in temperature results in a decrease in growth rate, the stress resistance of the cells could be improved. It has furthermore been shown that yeast in the stationary phase are better able to maintain their intracellular pH despite extracellular pH changes, than growing cells [42]. This could explain, why the postponed base addition resulted in an improved process performance.

### 3.3 Effects of submerse base addition on process performance

Although the earlier time point of base addition negatively impacted process performance, the rather severe effect of even minor pH amplitudes raises the question whether or not submerse addition of the base is harmful to the cells. Submerse addition has been suggested as a solution to improve mixing at large‐scale and thereby reduce occurring gradients [4, 25]. To assess its impact on the cells, 1‐CS studies, where base was either added through the reactor headspace or by submerse addition, were conducted.

**FIGURE 6:**
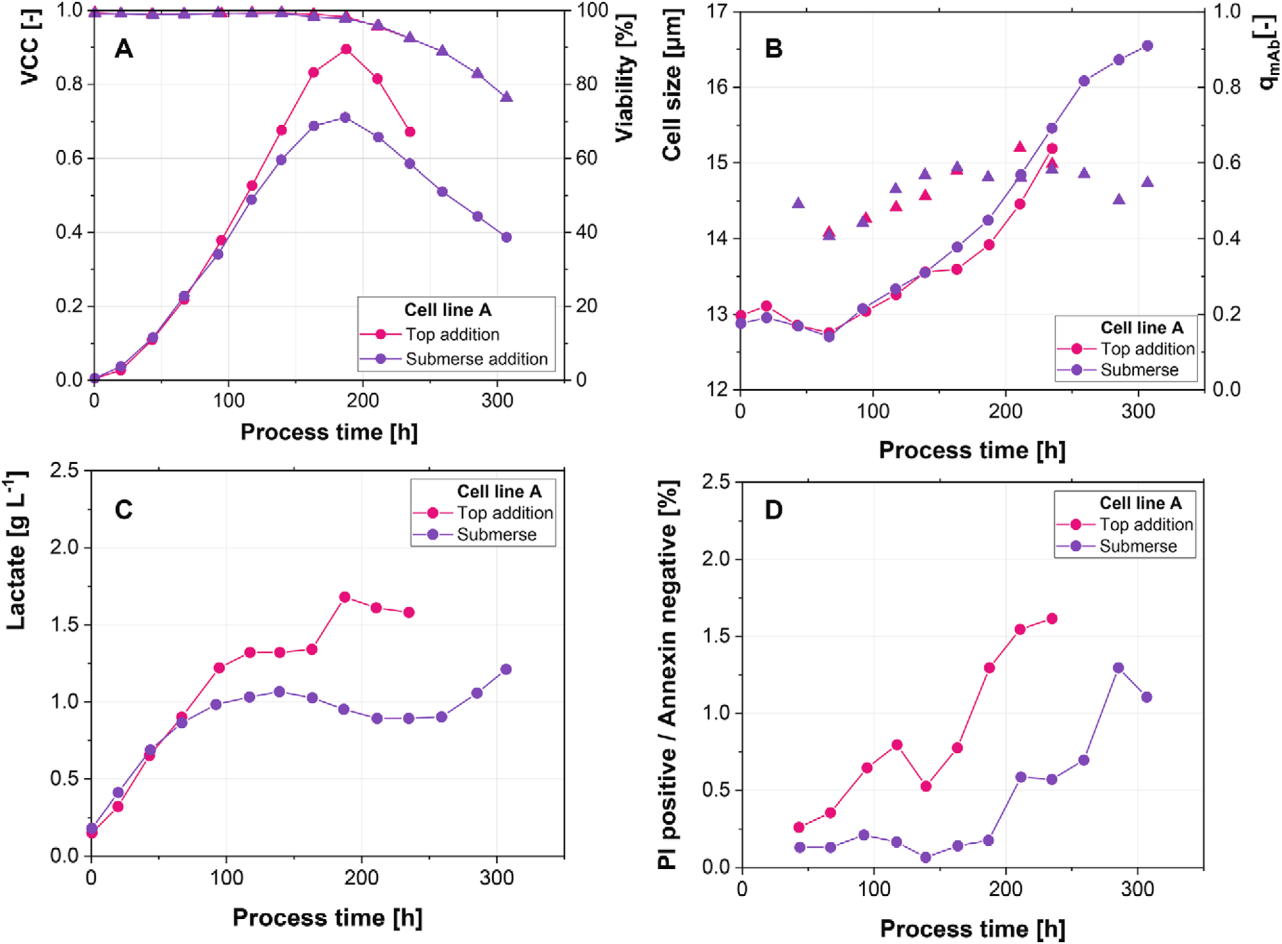
(A) Viable cell concentration (dots) and viability (triangles) trajectories. (B) Cell size (dots) and specific antibody production rate (triangles). (C) Absolute lactate levels. (D) Percentage of PI positive/Annexin negative cells for addition of base to the air‐liquid interface and submerse addition

Viabilities are similar in both cases, while the maximal VCC is 22% higher, when base is added from the top (Figure 6A). However, the VCC decline is steeper in this case. This is in good agreement with the increased levels of PI positive/Annexin negative particles, when base was added from the top of the reactor (Figure 6D). Furthermore, submerse addition of the base resulted in a more pronounced and earlier metabolic shift from lactate production to lactate consumption (Figure 6C). Overall, the only negative impact of submerse base addition is the decrease in maximal VCC. A possible cause for this decrease could be the continuous addition of base to the reactor during submerse addition. Since drops need to form when base is added through the reactor headspace, base is added stepwise rather than continuous, which might be beneficial. In context with these results, the observed decreased viable cell counts in response to the pH amplitudes are partially due to the submerse addition of the base. The decreased maximal VCC of cell line A for the small amplitudes (0.05 pH units) (Figure 2) can therefore be entirely explained by the submerse addition. Other responses, for example, the decreased qmAb and differences in cell size, are however not a result of the submerse base addition, but of the base addition strategy (Figure 6B).

## 4 CONCLUDING REMARKS

The aim of the study was to expose two different CHO cell lineages to reoccurring pH amplitudes, which they likely encounter in large‐scale bioreactors. The two investigated cell lines showed similar overall responses, like decreased VCC and mainly unaffected lactate levels, when they were exposed to small amplitudes (0.05 pH units) and when pH control was narrow (no pH amplitudes) in the 2‐CS. Differences were observed for the qmAb, where cell line B showed an increased productivity throughout the first half of the process, when cells were exposed to small (0.05 pH units) and medium amplitudes (0.1 pH units). In comparison to previously published work, where cell line A was exposed to pH amplitudes of up to 0.4 pH units, the maximal VCC was observed to be decreased further in response to the smaller amplitudes, as well as the narrow pH control (no pH amplitudes). A possible root cause for these observations is the earlier start of pH correction in the current study. Delaying the starting point of base addition to match the temperature shift slightly increased VCC and resulted in a more pronounced lactate metabolic shift. This is likely due to the inverse correlation between fast growth and adaptability of the cells. Therefore, base addition should be postponed as long as possible, at least to the point where growth is not exponential anymore. Particularly during the early stages, pH control could be accomplished by sparged gases only, which has been recently demonstrated as an alternative to base addition [43]. Submerse base addition, which has been proposed as a solution to minimize gradients, resulted in a decreased VCC and is therefore not an ideal strategy. Overall, process design, particularly in regards to the starting point of pH correction, impacts the influence, which pH inhomogeneity has on overall process performance. Therefore, the improvement of the process design can be an effective tool to minimize the negative effects of base addition.

## CONFLICT OF INTEREST

The authors have declared no conflict of interest.

## References

[1] Seymour, P. , Ecker, D. M. , Global biomanufacturing trends, capacity, and technology drivers: industry biomanufacturing capacity overview. Am. Pharm. Rev. 2016, 20, 1–8.

[2] Schmidt, F. R. , Optimization and scale up of industrial fermentation processes. Appl. Microbiol. Biotechnol. 2005, 68, 425–435. 16001256 10.1007/s00253-005-0003-0

[3] Xing, Z. , Kenty, B. M. , Li, Z. J. , Lee, S. S. , Scale‐up analysis for a CHO cell culture process in large‐scale bioreactors. Biotechnol. Bioeng. 2009, 103, 733–746. 19280669 10.1002/bit.22287

[4] Langheinrich, C. , Nienow, A. W. , Control of pH in large‐scale, free suspension animal cell bioreactors: alkali addition and pH excursions. Biotechnol. Bioeng. 1999, 66, 171–179. 10577471 10.1002/(sici)1097-0290(1999)66:3<171::aid-bit5>3.0.co;2-t

[5] Nienow, A. W. , Langheinrich, C. , Stevenson, N. C. , Nicholas Emery, A. et al. Homogenisation and oxygen transfer rates in large agitated and sparged animal cell bioreactors: some implications for growth and production. Cytotechnology. 1996, 22, 87–94. 22358918 10.1007/BF00353927

[6] Janakiraman, V. , Kwiatkowski, C. , Kshirsagar, R. , Ryll, T. , Huang, Y. M. , Application of high‐throughput mini‐bioreactor system for systematic scale‐down modeling, process characterization, and control strategy development. Biotechnol. Prog. 2015, 31, 1623–1632. 26317495 10.1002/btpr.2162

[7] Bertrand, V. , Vogg, S. , Villiger, T. K. , Stettler, M. et al. Proteomic analysis of micro‐scale bioreactors as scale‐down model for a mAb producing CHO industrial fed‐batch platform. J. Biotechnol. 2018, 279, 27–36. 29719200 10.1016/j.jbiotec.2018.04.015

[8] Yang, J.‐D. , Lu, C. , Stasny, B. , Henley, J. et al. Fed‐batch bioreactor process scale‐up from 3‐L to 2,500‐L scale for monoclonal antibody production from cell culture. Biotechnol. Bioeng. 2007, 98, 141–154. 17657776 10.1002/bit.21413

[9] Smelko, J. P. , Wiltberger, K. R. , Hickman, E. F. , Morris, B. J. et al. Performance of high intensity fed‐batch mammalian cell cultures in disposable bioreactor systems. Biotechnol. Prog. 2011, 27, 1358–1364. 21626722 10.1002/btpr.634

[10] Gramer, M. J. , Ogorzalek, T. , A semi‐empirical mathematical model useful for describing the relationship between carbon dioxide, pH, lactate and base in a bicarbonate‐buffered cell‐culture process. Biotechnol. Appl. Biochem. 2007, 47, 197–204. 17362203 10.1042/BA20070001

[11] Ahuja, S. , Jain, S. , Ram, K. , Application of multivariate analysis and mass transfer principles for refinement of a 3‐L bioreactor scale‐down model‐when shake flasks mimic 15,000‐L bioreactors better. Biotechnol. Prog. 2015, 31, 1370–1380. 26097232 10.1002/btpr.2134

[12] Xu, S. , Jiang, R. , Mueller, R. , Hoesli, N. et al. “Probing lactate metabolism variations in large‐scale bioreactors. Biotechnol. Prog. 2018, 34, 756–766. 29464875 10.1002/btpr.2620

[13] Brunner, M. , Doppler, P. , Klein, T. , Herwig, C. , Fricke, J. , Elevated pCO_2_ affects the lactate metabolic shift in CHO cell culture processes. Eng. Life Sci. 2018, 18, 204–214. 32624899 10.1002/elsc.201700131 PMC6999464

[14] Takuma, S. , Hirashima, C. , Piret, J. M. , Dependence on glucose limitation of the pCO_2_ influences on CHO cell growth, metabolism and IgG production. Biotechnol. Bioeng. 2007, 97, 1479–1488. 17318909 10.1002/bit.21376

[15] Kimura, R. , Miller, W. M. , Effects of elevated pCO_2_ and/or osmolality on the growth and recombinant tPA production of CHO cells. Biotechnol. Bioeng. 1996, 52, 152–160. 18629861 10.1002/(SICI)1097-0290(19961005)52:1<152::AID-BIT15>3.0.CO;2-Q

[16] Villiger, T. K. , Neunstoecklin, B. , Karst, D. J. , Lucas, E. et al. Experimental and CFD physical characterization of animal cell bioreactors: from micro‐ to production scale. Biochem. Eng. J. 2018, 131, 84–94.

[17] Jiang, R. , Chen, H. , Xu, S. , pH excursions impact CHO cell culture performance and antibody N‐linked glycosylation. Bioprocess Biosyst. Eng. 2018, 41, 1731–1741. 30088083 10.1007/s00449-018-1996-y

[18] Nienow, A. W. , Scott, W. H. , Hewitt, C. J. , Thomas, C. R. et al. Scale‐down studies for assessing the impact of different stress parameters on growth and product quality during animal cell culture. Chem. Eng. Res. Des. 2013, 91, 2265–2274.

[19] Brunner, M. , Braun, P. , Doppler, P. , Posch, C. et al. The impact of pH inhomogeneities on CHO cell physiology and fed‐batch process performance, two‐compartment scale‐down modelling and intracellular pH excursion. Biotechnol. J. 2017, 12, 1–13. 10.1002/biot.201600633 28078826

[20] Osman, J. J. , Birch, J. , Varley, J. , The response of GS‐NS0 myeloma cells to pH shifts and pH perturbations. Biotechnol. Bioeng. 2001, 75, 63–73. 11536128 10.1002/bit.1165

[21] Haringa, C. , Tang, W. , Wang, G. , Deshmukh, A. T. et al. Computational fluid dynamics simulation of an industrial P. chrysogenum fermentation with a coupled 9‐pool metabolic model: towards rational scale‐down and design optimization. Chem. Eng. Sci. 2018, 175, 12–24.

[22] Haringa, C. , Tang, W. , Deshmukh, A. T. , Xia, J. et al. Euler‐Lagrange computational fluid dynamics for (bio)reactor scale down: an analysis of organism lifelines. Eng. Life Sci. 2016, 16, 652–663. 27917102 10.1002/elsc.201600061 PMC5129516

[23] Paul, K. , Böttinger, K , Mitic, BM , et al. Development, characterization and application of a two‐Compartment system to investigate the impact of pH inhomogeneities in large‐scale CHO‐based processes. Eng Life Sci. 2020, 1–11. 10.1002/elsc.202000009. PMC7401239 32774209

[24] Reinhart, D. , Damjanovic, L. , Kaisermayer, C. , Sommeregger, W. et al. Bioprocessing of Recombinant CHO‐K1, CHO‐DG44, and CHO‐S: CHO expression hosts favor either mAb production or biomass synthesis. Biotechnol. J. 2019, 14, 1–11. 29701329 10.1002/biot.201700686

[25] Sieblist, C. , Jenzsch, M. , Pohlscheidt, M. , Lübbert, A. , Insights into large‐scale cell‐culture reactors: I. Liquid mixing and oxygen supply. Biotechnol. J, 2011, 6, 1532–1546. 21818860 10.1002/biot.201000408

[26] Kumar, N. , Borth, N. , Flow‐cytometry and cell sorting: an efficient approach to investigate productivity and cell physiology in mammalian cell factories. Methods. 2012, 56, 366–374. 22426008 10.1016/j.ymeth.2012.03.004

[27] Charaniya, S. , Le, H. , Rangwala, H. , Mills, K. et al. Mining manufacturing data for discovery of high productivity process characteristics. J. Biotechnol. 2010, 147, 186–197. 10.1016/j.jbiotec.2010.04.005 20416347

[28] Le, H. , Kabbur, S. , Pollastrini, L. , Sun, Z. et al. Multivariate analysis of cell culture bioprocess data—Lactate consumption as process indicator. J. Biotechnol. 2012, 162, 210–223. 22974585 10.1016/j.jbiotec.2012.08.021

[29] Kim, M. S. , Kim, N. S. , Sung, Y. H. , Lee, G. M. , Biphasic culture strategy based on hyperosmotic pressure for improved humanized antibody production in Chinese hamster ovary cell culture. In Vitro Cell. Dev. Biol. Anim. 2002, 38, 314–319. 12513118 10.1290/1071-2690(2002)038<0314:BCSBOH>2.0.CO;2

[30] Pfizenmaier, J. , Junghans, L. , Teleki, A. , Takors, R. , Hyperosmotic stimulus study discloses benefits in ATP supply and reveals miRNA/mRNA targets to improve recombinant protein production of CHO cells. Biotechnol. J. 2016, 11, 1037–1047. 27214792 10.1002/biot.201500606

[31] Fomina‐Yadlin, D. , Du, Z. , McGrew, J. T. , Gene expression measurements normalized to cell number reveal large scale differences due to cell size changes, transcriptional amplification and transcriptional repression in CHO cells. J. Biotechnol. 2014, 189, 58–69. 25194670 10.1016/j.jbiotec.2014.08.037

[32] Lloyd, D. R. , Holmes, P. , Jackson, L. P. , Emery, A. N. , Al‐Rubeai, M. , Relationship between cell size, cell cycle and specific recombinant protein productivity. Cytotechnology 2000, 34, 59–70. 19003381 10.1023/A:1008103730027 PMC3449736

[33] Kim, T. K. , Chung, J. Y. , Sung, Y. H. , Lee, G. M. , Relationship between cell size and specific thrombopoietin productivity in Chinese hamster ovary cells during dihydrofolate reductase‐mediated gene amplification. Biotechnol. Bioprocess Eng. 2001, 6, 332–336.

[34] Pan, X. , Dalm, C. , Wijffels, R. H. , Martens, D. E. , Metabolic characterization of a CHO cell size increase phase in fed‐batch cultures. Appl. Microbiol. Biotechnol. 2017, 101, 8101–8113. 28951949 10.1007/s00253-017-8531-y PMC5656727

[35] Han, Y. K. , Kim, Y. G. , Kim, J. Y. , Lee, G. M. , Hyperosmotic stress induces autophagy and apoptosis in recombinant chinese hamster ovary cell culture. Biotechnol. Bioeng. 2010, 105, 1187–1192. 20014438 10.1002/bit.22643

[36] Han, Y. K. , Ha, T. K. , Lee, S. J. , Lee, J. S. et al. Autophagy and apoptosis of recombinant Chinese hamster ovary cells during fed‐batch culture: effect of nutrient supplementation. Biotechnol. Bioeng. 2011, 108, 2182–2192. 21495016 10.1002/bit.23165

[37] Imamoto, Y. , Tanaka, H. , Takahashi, K. , Konno, Y. et al. Advantages of AlaGln as an additive to cell culture medium: use with anti‐CD20 chimeric antibody‐producing POTELLIGENT CHO cell lines. Cytotechnology 2013, 65, 135–143. 22695857 10.1007/s10616-012-9468-8 PMC3536879

[38] Van Engeland, M. , Nieland, L. J. W. , Ramaekers, F. C. S. , Schutte, B. et al. Annexin V‐affinity assay: a review on an apoptosis detection system based on phosphatidylserine exposure. Cytometry. 1998, 31, 1–9. 9450519 10.1002/(sici)1097-0320(19980101)31:1<1::aid-cyto1>3.0.co;2-r

[39] Vis, D. J. , Zakrzewska, A. , van Eikenhorst, G. , Burggraaff, J. E. C. et al. Genome‐wide analysis of yeast stress survival and tolerance acquisition to analyze the central trade‐off between growth rate and cellular robustness. Mol. Biol. Cell. 2011, 22, 4435–4446. 21965291 10.1091/mbc.E10-08-0721 PMC3216668

[40] Delvigne, F. , Baert, J. , Gofflot, S. , Lejeune, A. et al. Dynamic single‐cell analysis of Saccharomyces cerevisiae under process perturbation: comparison of different methods for monitoring the intensity of population heterogeneity. J. Chem. Technol. Biotechnol. 2015, 90, 314–323.

[41] Hallsworth, J. E. , Stress‐free microbes lack vitality. Fungal Biol. 2018, 122, 379–385. 29801781 10.1016/j.funbio.2018.04.003

[42] Valli, M. , Sauer, M. , Branduardi, P. , Borth, N. et al. Intracellular pH distribution in Saccharomyces cerevisiae cell populations, analyzed by flow cytometry. Appl. Environ. Microbiol. 2005, 71, 1515–1521. 15746355 10.1128/AEM.71.3.1515-1521.2005 PMC1065191

[43] Hoshan, L. , Jiang, R. , Moroney, J. , Bui, A. et al. Effective bioreactor pH control using only sparging gases. Biotechnol. Prog. 2019, 35, 1–7. 10.1002/btpr.2743 30421525

